# Mechanical Performance of Rat, Mouse and Mole Spring Traps, and Possible Implications for Welfare Performance

**DOI:** 10.1371/journal.pone.0039334

**Published:** 2012-06-29

**Authors:** Sandra E. Baker, Stephen A. Ellwood, Vito L. Tagarielli, David W. Macdonald

**Affiliations:** 1 Wildlife Conservation Research Unit, Department of Zoology, University of Oxford, Oxford, United Kingdom; 2 Department of Aeronautics, Imperial College, London, United Kingdom; Université Pierre et Marie Curie, France

## Abstract

Lethal spring traps are widely used for killing small mammals in the UK. Many require government approval, based primarily on humaneness. However, mole traps and break-back traps for rats and mice are exempt; those available vary widely in price and apparent quality. The EU is considering implementing a Trapping Directive that would alter UK legislation, and a recent report advised the EU that trapping legislation should cover all trapped species and encourage improvement of traps. Mechanical trap performance is often used as an indicator of welfare impact. We examined the mechanical evidence for scope to improve the welfare standards of rat, mouse and mole spring traps. We measured mechanical performance among a range of rat, mouse and mole traps. Impact momentum values varied 6-8 fold, and clamping force values 4-5.5 fold, among traps for killing each species. There was considerable overlap in the performance of rat and mouse traps. Trap-opening angle and spring type were related to impact momentum and clamping force in traps for both species. There was no relationship between price and mechanical performance in traps for any species, except talpa mole traps. We are unable to judge the direct welfare impact of the traps tested, but rather the potential welfare threat associated with their exemption from approval. The wide variation in mechanical performance in traps for each species, overlap in performance between rat and mouse traps and increasing availability of weaker plastic rodent traps indicate considerable scope for improving the humaneness of spring traps for rats, mice and moles. We conclude that all such traps should be subject to the UK approval process. New welfare categories might improve trap standards further. Our results could also help improve rodent trap design and assist consumers in selecting more powerful traps. Many thousands of rats, mice and moles might benefit.

## Introduction

Spring traps are widely used for trapping small mammals in the UK (e.g. see The Spring Traps Approval (England) Order 2012 at http://www.legislation.gov.uk/uksi/2012/13/schedule/made). In 1951, The Committee on Cruelty to Wild Animals produced a report in which they made important recommendations regarding spring traps, including that: “*It should be made illegal for any spring trap to be used, the design of which has not been approved by the Minister of Agriculture and Fisheries and the Secretary of State for Scotland, and those Ministers should approve only spring traps which will catch and kill wild animals without causing them unnecessary suffering*” [1]; see also http://hansard.millbanksystems.com/lords/1951/nov/28/spring-traps-bill-hl). In their report, the Committee also stated that “*the rat is regarded as one of the greatest animal pests…It is also a menace to public health…For these reasons its control and destruction are essential…*”. They concluded that break-back traps for use with rats and mice did not involve any unnecessary suffering. On mole trapping they said “…*We have had no evidence that* [mole] *trapping causes unnecessary suffering, except that one organisation mentioned that they had been given to understand that the spring of the ordinary type of mole-trap* [it is not clear which] *was too weak to kill instantaneously*”. As a result, The Committee concluded that it was not necessary to make any special recommendations regarding practices involving moles.

In 1954, The Pests Act gave effect to the Committee’s recommendations, making it an offence, *inter alia,* to use a spring trap for the purposes of killing or taking animals in England, Wales and Scotland, other than one approved by an Order of the Secretary of State. [NB The Act also banned gin traps on welfare grounds following considerable public agitation regarding their use [2].] Following the Committee’s comments regarding rats, mice and moles, The Small Ground Vermin Traps Order 1958 implemented a provision in The Pests Act to exempt from the approval process: (1) *“Spring traps known as break-back traps and commonly used for the destruction of rats, mice or other small ground vermin”*; and (2) *“Spring traps of the kind commonly used for catching moles in their runs”*. These exemptions persist today. Consequently, anyone can make and sell any such devices and those available on the market vary widely in price and apparent quality. [NB This exemption does not relate to other spring traps designed for catching rats or mice, e.g. certain BMI Magnum traps, Fenn traps etc, which do require approval and are not included in this study (http://www.legislation.gov.uk/uksi/2012/13/schedule/made).].

The approval of traps is a devolved issue but, under current English legislation, spring traps require approval under The Spring Traps Approval (England) Order 2012. Equivalent Orders have been introduced elsewhere in the UK. The humaneness of a spring trap is the main criterion upon which approval decisions are made [3].

Impact momentum and clamping force are widely accepted as indicators of welfare performance among spring traps internationally [4] and in Europe [5], and in certain circumstances these mechanical measures continue to be used as part of the formal approvals process in England (Department for Environment, Food and Rural Affairs (Defra), Pers. Comm.). For example, while the current assessment criteria state that new spring traps submitted to Defra for approval should be subjected to killing tests on free-moving animals (in captivity) (these criteria are based on the Agreement on International Humane Trapping Standards (http://www.canadainternational.gc.ca/eu-ue/assets/pdfs/eu25-en.pdf)), traps deemed equivalent in all relevant respects (e.g. “*in construction, in materials, in impact force or momentum, and in all other respects which are relevant to its effect or manner of operation as a trap”*) to one with existing approval are considered approved, e.g. by virtue of s. 2(1) (b) of The Spring Traps Approval (England) Order 2012, and so are effectively approved without testing. In some cases it may be considered possible to assess a new trap without using live animals, or it may not be practical to conduct sufficient suitable tests on live individuals of a particular species. In such cases Defra would need to consider whether other tests, such as mechanical tests of impact momentum and clamping force, were sufficient to make an assessment. Or it might be considered sufficient to use another source of available data, such as a manufacturer’s own test data if they can be judged as reliable. While new types of spring trap are likely to undergo killing tests on free-moving animals (rather than relying solely on mechanical tests), if the design allows, traps will first be subjected to mechanical tests to identify trap types which are unlikely to pass killing tests. Such traps would be recommended for refusal and would not proceed to live animal tests (Defra, Pers. Comm.).

Most spring trap use in Britain is arguably targeted against rats and mice. And, following the 2006 withdrawal of strychnine poison for controlling moles, spring traps are now the most popular method of mole control among British farmers and amenity managers (Baker et al, unpublished data). Given the scale of use of trapping with rats, mice and moles, the wide range of spring traps available for killing them and doubt expressed regarding the humaneness of at least some of these traps [6], [7], it seems questionable that spring traps for use with these species should continue to be exempt from approval. This exemption is an inconsistency that could have implications for the welfare of many thousands of animals each year.

For over a decade the EU has been considering the implementation of an EU Trapping Directive, which would set new standards for the approval and use of traps for wildlife management in Europe. One of the issues they are thought to be considering is which species are covered. Britain would have to comply with any regulations that such a Directive might make, potentially extending the existing legislation to cover more species (e.g. potentially moles, rats and mice) and more types of trap. In 2008 the EU commissioned a report from the Food and Environment Research Agency (FERA), on trapping standards, and examining options for such a Directive. The report was released by the EU in September 2011, and this concluded that “*any new (trapping) measures adopted by the (EU) Member States should cover all species that can legally be trapped because there is no scientific justification for not including all species*”. The report also recommended that measures should be taken to improve the standard of traps that are approved for use by introducing a tiered welfare category system [5].

Our overall aim was to examine the evidence, based on variation in mechanical performance, that there is scope to improve the welfare standard of rat and mouse break-back traps, and mole spring traps, available in the UK. We conducted mechanical tests on a range of break-back traps, and on three commonly-used types of mole trap (scissors, Duffus, talpa) from a number of different manufacturers. We predicted that mechanical performance would vary widely among traps intended for the same purpose, e.g. mouse traps, rat traps, and each type of mole trap. We also predicted that mechanical performance would vary among the three types of mole trap. Our predictions were met, indicating that there may be considerable scope for improving the welfare standard of break-back traps and mole spring traps available in the UK.

## Methods

### Sourcing Rat and Mouse Traps

We identified 23 brands of mouse, and 18 brands of rat, break-back traps from shops and web-based sources in the UK (Table S1 and Figure S1), and purchased one trap of each type (or the minimum number that could be purchased where they were only available in bulk). Traps were purchased as cheaply as possible, where there was a choice of supplier, and the unit prices recorded. Trap bodies were made of wood, plastic or metal with striking bars and components of either plastic or metal. Traps varied in terms of the trap-opening angle, as measured when in the set position, and in spring type. Spring types were classified as peg (PEG), double peg (DPEG), jaw (JAW), or pull (PULL) type (Figure S2A-D), and trap-opening angles were measured for each of the 41 trap types (Figure S3). From the trap types purchased we identified six types of mouse trap and six types of rat trap that we considered to represent the range of the various trap features (opening angle, spring type, materials) and we purchased a further 14 replicates of each. These trap types made up the ‘replicated-set’ (Table 1). The total set of traps consisted of 209 traps (102 rat traps and 107 mouse traps) (see Table S2).

**Table 1:** Mouse and rat traps selected for the replicated set, and their features. A) mouse traps; B) rat traps.

A) Mouse traps	Angle category	Angle (degrees)	Spring type	Body material	Bar material
Ma	1	45	DPEG	P	P
Mb	1	60	PULL	P	P
Mc	1	70	JAW	P	P
Md	1	80	DPEG	P	M
Me	3	180	PEG	W	M
Mf	3	180	PEG	W	M
**B) Rat traps**	**Angle category**	**Angle (degrees)**	**Spring type**	**Body material**	**Bar material**
Ra	1	70	JAW	P	P
Rb	1	70	DPEG	P	P
Rc	1	80	DPEG	P	M
Rd	3	180	DPEG	M	M
Re	3	180	PEG	W	M
Rf	3	180	DPEG	W	M

Angles are trap-opening angle in degrees. Angle categories are: category 1, 45-89 degrees; category 2, 90-134 degrees; category 3, 135-180 degrees. Materials are either: P = plastic; W = wood; or M = metal.

### Sourcing Mole Traps

We identified mole trap brands of the three main spring trap types available: scissors, Duffus and talpa (see Figure S4A-C). Scissors traps were sometimes marketed as ‘claw’ traps, Duffus traps as ‘tunnel’ traps, and talpa traps as ‘Talpex-style’ and ‘spring’ traps. We found six major brands which appeared most conspicuous in the market. Three brands produced all three trap types (Trapman™, Mole Traps UK™ and Holey Moley™), two further brands produced scissors and Duffus-style traps (Procter Pest Stop™ and The Big Cheese™), and one produced only talpa-style traps (Talpex™). We bought 20 replicates of each of the 14 manufacturer/trap type combinations, a total of 280 traps, in order to examine manufacturer and trap type effects. The traps were bought as cheaply as possible and unit prices recorded.

### Mechanical Measurements

We devised bespoke systems for measuring both the static clamping force 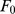 and the dynamic force versus time series 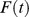
exerted by different traps at selected trap-openings, representative of the size of target animals. The dynamic force versus time series were integrated in time, in order to calculate the trap impulse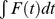; this is equal to the equivalent linear momentum (impact momentum) possessed by the moving part of the traps at the selected trap-opening by Newton’s Second Law [8], p128, Section 3.4).
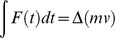



Static clamping forces were measured using a resistive load cell (R.D.P. Electronics Ltd, Sole UK; model 31; Sensitivity = 16.54 mV/N) with a 10V DC excitation. The load cell was physically clamped between the striking elements of the trap such that force was measured directly. Load cell output was amplified (by a Fylde, 351UA amplifier of gain = 1000) and the amplified signal was recorded by an oscilloscope (Tektronix; model DPO 3014). The load cell calibration factors were used to convert the amplifier’s output voltage to clamping force (in Newtons (N)).

Dynamic force histories were measured using a piezoelectric load cell (Omega Technologies Ltd, UK; model DLC101-500; Sensitivity = 2.383 mV/N). Traps were triggered so that the dynamic load cell was caught between the striking elements, as an animal would be, and the measured dynamic force versus time histories were employed to calculate the impact momentum of traps at selected openings, as described above.

The measured clamping force and impact momentum were independent of the load cell type and depended only on the trap mechanics. Both load cells were supplied with their own calibration certificates, and were calibrated in the lab to confirm the figures therein. The test jig was designed to have minimum mass, in order to ensure that inertia forces associated with the jig’s moving parts were negligible compared to impact forces.

#### Rat and mouse traps

We estimated the diameter of a mouse and a rat at a likely point of capture (immediately behind the forelegs) as 20 mm and 40 mm respectively and adapted the load cells accordingly, using aluminium ‘spacers’, so that static and dynamic measurements were taken at an aperture appropriate for the species concerned. These estimates were based on reports in the literature [9] and, for mice, post-mortem measurements supplied by the Vet Services Department at the University of Oxford.

Clamping forces were measured first. Before making measurements we stretched each trap to its fully open position (as if to set it), and measured clamping force post-stretch. Clamping force was measured for every trap, including those in the non-replicated set and all individual traps in the replicated set. Impact momentum was recorded for all traps in the non-replicated set and 10 of the 15 traps of each type in the replicated set. Impact momentum could be measured for only 17 types of rat trap because one of the non-replicated set broke during dynamic testing; therefore n = 17 for impact momentum tests on rat traps. Clamping force and impact momentum were each measured five times on one trap (trapID1) of each type; these were averaged to produce mean values of the clamping force and impact momentum for each trap type for inclusion in analysis.

#### Mole traps

We used the same apparatus to record clamping force and impact momentum for mole traps, but with one exception: the dynamic load cell was mounted within an aluminium jig (Figure S5). The jig protected the dynamic load cell from non-axial strikes, which could cause incorrect measurements and damage to the load cells (non-axial strikes were possible when testing the three types of mole trap). We assumed the diameter of a mole to be 40 mm at the likely point of capture (thorax, thorax/abdomen or abdomen (Baker et al, unpublished data)), as reported by Atkinson et al. [10], and adapted the load-cells using the spacers described for testing rat traps. Clamping force was measured for all 20 mole traps of each trap type/manufacturer combination and impact momentum recorded for 10 of the 20 traps of each.

### Data analysis

We examined differences in the mechanical performance of mouse and rat traps and scrutinised variability among traps for each species. We also investigated variability in performance between trap types in the replicated set. Then we tested the effect of trap-opening angle and spring type on rat and mouse trap performance. We examined differences in the mechanical performance of mole traps of different types, and made by different manufacturers, as well as variability in performance within trap type/manufacturer combination. (Mole trap manufacturer was treated as a fixed effect because we were interested in testing for differences between these key manufacturers.) We also investigated whether unit price was a useful predictor of trap performance in rat, mouse or mole traps. Responses were log transformed before statistical analysis using SAS software [11]. Trap brands are anonymised in the results.

## Results

### Variability Among Trap Types in Rat and Mouse Traps

**Figure 1:**
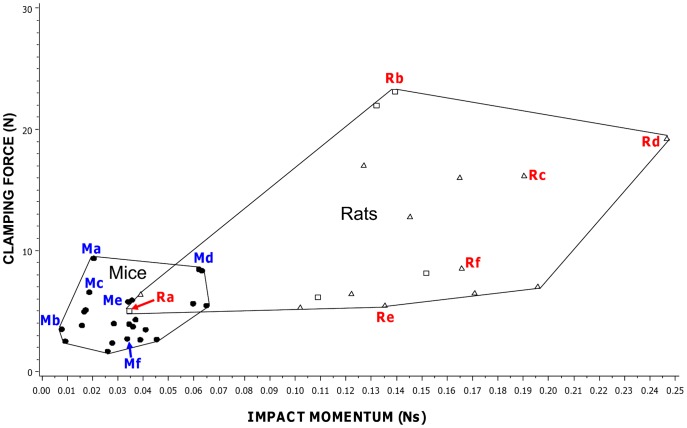
Impact momentum against clamping force for mouse and rat traps. Mouse trap types (n = 23) are represented by circles and rat trap types (n = 17) by triangles or squares. Each point represents a different trap type and is the mean of five measurements on one trap. [The raw data from which these were calculated are shown in Figure S6.] Points marked Ma-f (labelled blue) and Ra-f (labelled red) are trap types in the mouse and rat replicated sets respectively, with features shown in Table 1.

Both impact momentum and clamping force varied widely among the mouse and rat traps assessed (Figure 1). Impact momentum varied between 0.01 and 0.06 Ns (mean = 0.03, SE = 0.003, n = 23) for mouse traps and between 0.03 and 0.25 Ns (mean = 0.14, SE = 0.01, n = 17) for rat traps. Clamping force varied between 1.69 and 9.36 N (mean = 4.64, SE = 0.43, n = 23) and between 5.03 and 23.10 N (mean = 11.32, SE = 1.45, n = 18) for mouse and rat traps respectively. Impact momentum varied by a factor of 6 for mouse traps and 8 for rat traps. Clamping force varied by a factor of approximately 5.5 for mouse traps and 4.5 for rat traps.

There was strong evidence for differences between mouse and rat traps in both measures (impact momentum F_1,38_ = 69.38, P<0.001; clamping force F_1,39_ = 30.92, P<0.001). Despite this overall difference between mouse and rat traps, there was considerable overlap between traps for the two species in both impact momentum (13 mouse and 2 rat traps in overlap) and clamping force (9 mouse and 10 rat traps in overlap) (Figure 1).

**Figure 2:**
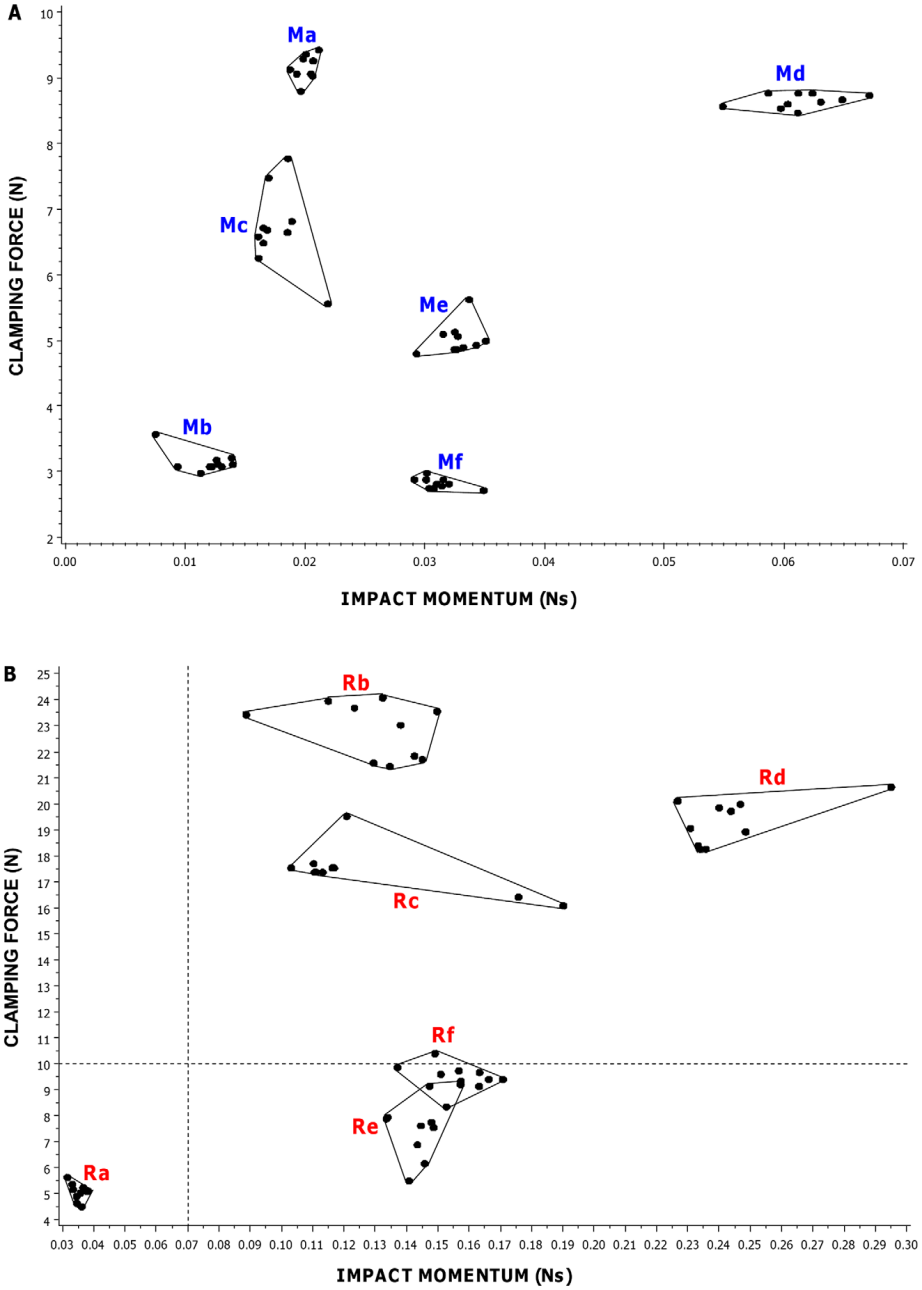
Raw data for impact momentum against clamping force in mouse and rat trap replicated sets. A) mouse trap types; B) rat trap types. Each point represents an individual trap and traps of the same type are enclosed within a polygon. These trap types are identified on Figure 1. For scale, the small square, demarcated by the dotted lines and the axes on Figure 2B, represents the entire range of clamping forces and impact momentums shown by Figure 2A.

Using data from the replicated trap sets, we examined variation in both impact momentum and clamping force among six types of mouse trap and among six types of rat trap. Both measures differed among mouse trap types (impact momentum F_5,54_ = 360.22, P<0.001; clamping force F_5,84_ = 1751.89, P<0.001) and rat trap types (impact momentum F_5,54_ = 307.57, P<0.001; clamping force F_5,84_ = 803.11, P<0.001). See Figures 2A-B. The spread of measurements for all rat and mouse trap types (not just the replicated sets) is shown in Figure S6.

### Effect of Spring Type and Trap-opening Angle on Trap Performance in Rat and Mouse Traps

Trap-opening angles for mouse traps ranged between 45-180 degrees, and for rat traps ranged between 70-180 degrees. Trap types were allocated to one of three opening angle categories covering the full range of angles represented (category 1, 45-89 degrees; category 2, 90-134 degrees; category 3, 135-180 degrees). All traps in category 3 had an opening angle of approximately 180 degrees and had wooden or metal bodies and a metal striking bar, with the exception of one trap which had a plastic body. All traps in categories 1 and 2 had plastic bodies and either plastic or metal striking components.

JAW springs were found in angle category 1 traps, PULL springs in categories 1 and 2, PEG springs in category 3 and DPEG springs in all three angle categories (Table S3). Mouse traps were represented by all three angle categories and all four spring types, while rat traps included all spring types and were in either angle category 1 or 3. Because spring types were represented differently across angle categories we were unable to test for interactions among spring type and angle category. Because the numbers of mouse and rat trap types were different across the spring type and angle category combinations, we examined the effects of angle and spring type separately for mouse and rat traps, and subsets of the data where possible.

#### Rat traps

There was an overall tendency for rat traps with a greater opening angle to produce a greater impact momentum (Table 2A), although this effect was not statistically significant (F_1,15_ = 2.22, P = 0.157). Among traps with a DPEG spring there was a similar but non significant pattern (F_1,8_ = 0.62, P = 0.455; Table 2B). Overall, rat trap impact momentum was affected by spring type (F_3,13_ = 24.17, P<0.001), with DPEGs producing the greatest impact momentum, followed by PULL, PEG and JAW springs (Table 2B). There was a similar pattern among spring types for traps in angle category 1 (F_2,5_ = 59.65, P<0.001), and a similar but non statistically-significant pattern among spring types for traps in angle category 3 (F_1,7_ = 2.45, P = 0.161) (Table 2A).

**Table 2:** Mean values and standard errors of impact momentum (Ns) among rat trap types*.

A) Angle	n	Mean	SE	Spring	N	Mean	SE
Angle 1	8	0.12	0.02	DPEG	5	0.15	0.01
				PULL	1	0.15	.
				JAW	2	0.04	0.00
Angle 3	9	0.16	0.02	DPEG	5	0.18	0.02
				PEG	4	0.13	0.01
**B) Spring**	**n**	**Mean**	**SE**	**Angle**	**N**	**Mean**	**SE**
DPEG	10	0.16	0.01	Angle 1	5	0.15	0.01
				Angle 3	5	0.18	0.02
PULL	1	0.15	.	Angle 1	1	0.15	.
PEG	4	0.13	0.01	Angle 3	4	0.13	0.01
JAW	2	0.04	0.00	Angle 1	2	0.04	0.00

* These are shown by: A) angle category; and B) spring type. Impact momentum values are the mean of five measurements on one trap.

In contrast to impact momentum, clamping force was significantly greater for rat traps with a smaller opening angle (F_1,16_ = 5.42, P = 0.033; Table 3A) describing an inverse linear relationship (F_1,8_ = 12.41, P = 0.008); where log (mean clamping force (N))  = 3.50949– (0.00773× opening angle). Among DPEG springs, the clamping force was also greater where the opening angle was smaller (F = 11.57_1,8_, P = 0.009; Table 3B). Overall, rat trap clamping forces were greater for DPEG springs followed by those with PULL, PEG and JAW springs (Table 3B); however this was not statistically significant (F_3,14_ = 2.47, P = 0.105), probably because the effects of spring type and angle were structurally confounded. However, among traps in angle category 1, clamping force did vary significantly with spring type (F_2,5_ = 32.73, P = 0.001; Table 3A), with DPEGs producing the greatest clamping force, followed by PULL and JAW springs. Among traps in angle category 3, DPEG springs also produced the greater clamping force, but this was not statistically significant (F_1,8_ = 0.24, P = 0.635) (Table 3A).

**Table 3:** Mean values and standard errors of clamping force (N) among rat trap types*.

A) Angle	n	Mean	SE	Spring	N	Mean	SE
Angle 1	8	14.83	2.31	DPEG	5	18.87	1.52
				PULL	1	12.82	.
				JAW	2	5.73	0.70
Angle 3	10	8.51	1.35	DPEG	5	9.36	2.53
				PEG	5	7.66	1.19
**B) Spring**	**n**	**Mean**	**SE**	**Angle**	**N**	**Mean**	**SE**
DPEG	10	14.11	2.11	Angle 1	5	18.87	1.52
				Angle 3	5	9.36	2.53
PULL	1	12.82	.	Angle 1	1	12.82	.
PEG	5	7.66	1.19	Angle 3	5	7.66	1.19
JAW	2	5.73	0.70	Angle 1	2	5.73	0.70

* These are shown by: A) angle category; and B) spring type. Clamping force values are the mean of five measurements on one trap.

#### Mouse traps

Overall, the impact momentum produced by mouse traps was significantly related to trap-opening angle (F_2,20_ = 5.16, P = 0.016), with the greatest impact momentum being associated with traps in angle category 2 (Table 4A). Among traps with a DPEG spring, there was no evidence for variation in impact momentum across the three angle categories (F_2,6_ = 1.19, P = 0.368). Traps with a PULL spring were represented only in angle categories 1 and 2, and the impact momentum produced by these traps was significantly greater in the larger angle category (F_1,1_ = 367.09_,_ P = 0.033; Table 4B). The trend in impact momentums produced by mouse traps with different spring types was similar to that observed among rat traps, with the greatest impact momentums produced by DPEG springs, followed by PULL/PEG and JAW (Table 4B), although this was not statistically significant (F_3,19_ = 2.69, P = 0.076). Again, as with rat traps, this pattern was reflected within each angle category (Table 4A). This effect was statistically significant for angle category 2 (F_1,2_ = 30.49, P = 0.031) but not categories 1 and 3 (F_2,7_ = 3.47, P = 0.090; F_1,7_ = 1.91, P = 0.210).

**Table 4:** Mean values and standard errors of impact momentum (Ns) among mouse trap types*.

A) Angle	N	Mean	SE	Spring	N	Mean	SE
Angle 1	10	0.03	0.01	DPEG	6	0.04	0.01
				PULL	1	0.01	.
				JAW	3	0.01	0.00
Angle 2	4	0.05	0.01	DPEG	2	0.06	0.00
				PULL	2	0.04	0.00
Angle 3	9	0.03	0.00	DPEG	1	0.04	.
				PEG	8	0.03	0.00
**B) Spring**	**N**	**Mean**	**SE**	**Angle**	**N**	**Mean**	**SE**
DPEG	9	0.04	0.01	Angle 1	6	0.04	0.01
				Angle 2	2	0.06	0.00
				Angle 3	1	0.04	.
PULL	3	0.03	0.01	Angle 1	1	0.01	.
				Angle 2	2	0.04	0.00
PEG	8	0.03	0.00	Angle 3	8	0.03	0.00
JAW	3	0.01	0.00	Angle 1	3	0.01	0.00

* These are shown by: A) angle category; and B) spring type. Impact momentum values are the mean of five measurements on one trap.

As for rat traps, the clamping force produced by mouse traps was greater for traps with a smaller opening angle (F_2,20_ = 3.91, P = 0.037; Table 5A) and there was an inverse linear relationship between angle and clamping force (F_1,7_ = 3.91, P = 0.088); where log (mean clamping force (N))  = 2.22026– (0.00589× opening angle). Clamping force also increased as angle category decreased among traps with DPEG springs and among those with PULL springs, but neither was statistically significant (DPEG, F_2,6_ = 3.41, P = 0.103; PULL, F_1,1_ = 0.39, P = 0.644; Table 5B). Overall, the clamping forces produced by mouse traps were affected by spring type, with forces being greatest for those with DPEG springs followed by those with JAW, PEG and then PULL springs (F_3,19_ = 3.18, P = 0.047; Table 5B). A similar pattern was observed among traps in angle categories 1 and 2 (Table 5A); this was significant for angle category 2 (F_1,2_ = 20.39, P = 0.046) but not category 1 (F_2,7_ = 1.70, P = 0.250). While the clamping force produced by the only trap in angle category 3 with a DPEG spring was smaller than the mean for the eight in that category with PEG springs, this was not a significant effect (F_1,7_ = 0.34, P = 0.580).

**Table 5:** Mean values and standard errors of clamping force (N) among mouse trap types*.

A) Angle	N	Mean	SE	Spring	N	Mean	SE
Angle 1	10	5.85	0.73	DPEG	6	6.83	0.90
				JAW	3	4.68	1.18
				PULL	1	3.51	.
Angle 2	4	4.30	0.74	DPEG	2	5.54	0.08
				PULL	2	3.06	0.40
Angle 3	9	3.46	0.41	DPEG	1	2.63	.
				PEG	8	3.56	0.45
**B) Spring**	**N**	**Mean**	**SE**	**Angle**	**N**	**Mean**	**SE**
DPEG	9	6.07	0.75	Angle 1	6	6.83	0.90
				Angle 2	2	5.54	0.08
				Angle 3	1	2.63	.
JAW	3	4.68	1.18	Angle 1	3	4.68	1.18
PEG	8	3.56	0.45	Angle 3	8	3.56	0.45
PULL	3	3.21	0.28	Angle 1	1	3.51	.
				Angle 2	2	3.06	0.40

* These are shown by: A) angle category; and B) spring type. Clamping force values are the mean of five measurements on one trap.

### Relationship between Trap Performance and Price in Rat and Mouse Traps

Different types of mouse trap cost £0.26-£3.45 each (mean = £1.67, SE = £0.15, n = 23). Different types of rat trap cost £1.87-£6.99 (mean = £3.91, SE = £0.38, n = 18). There was no evidence of a simple linear relationship between price and trap performance for either mouse traps (impact momentum, F_1,7_ = 0.30, P = 0.598; clamping force, F_1,7_ = 0.47, P = 0.515) or rat traps (impact momentum, F_1,8_ = 0.23, P = 0.641; clamping force, F_1,8_ = 3.18, P = 0.112). Among mouse traps, those with the greatest and smallest impact momentums cost £2.00 and £1.99 respectively (both above the mean mouse trap price of £1.67), while those with the greatest and smallest clamping forces cost £1.50 and £1.00 (both below the mean). Among rat traps, that with the greatest impact momentum cost £2.49 (below the mean rat trap price of £3.91) and that with the greatest clamping force cost £4.98 (above the mean). The worst performer, with both the smallest impact momentum and smallest clamping force, cost £4.99 (above the mean price).

### Variability Among Trap Types and Manufacturers in Mole Traps

**Figure 3:**
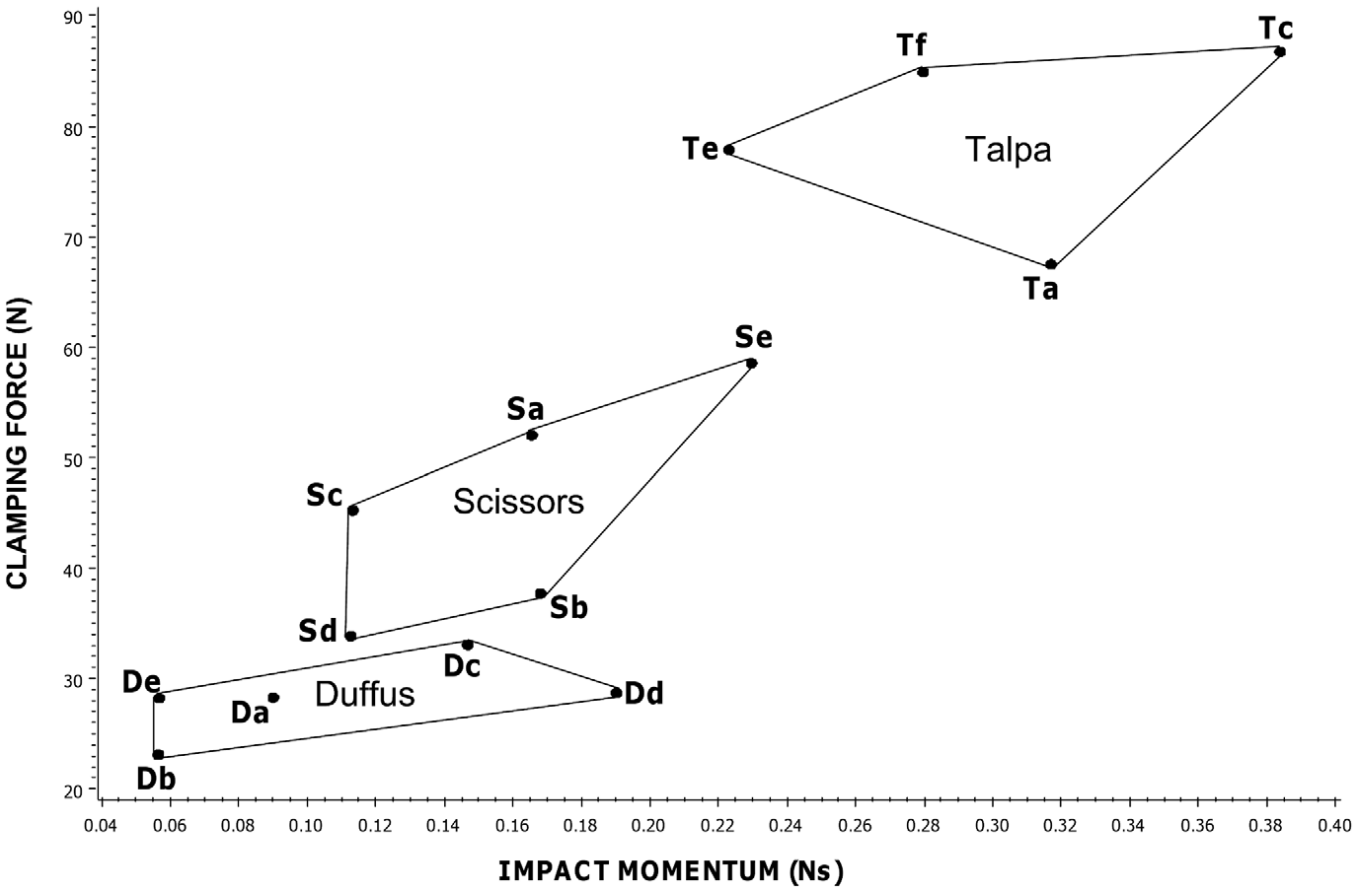
Impact momentum against clamping force for mole traps. Each point represents a different trap type/manufacturer combination and is the mean of measurements on 10 individual traps for impact momentum and 20 traps for clamping force. Points for traps of the same type but from different manufacturers are enclosed within a polygon. Points marked Da-e represent Duffus traps (n = 5), those marked Sa-e represent scissors traps (n = 5) and those marked Ta,c,e,f represent talpa-style traps (n = 4). Letters a-f represent different manufacturers.

Impact momentum and clamping force varied widely among mole trap types, and among manufacturers of the same trap type (Figure 3). Impact momentum varied between 0.06 and 0.19 Ns (mean = 0.11, SE = 0.03, n = 5) for Duffus traps, 0.11 and 0.23 Ns (mean 0.16, SE = 0.02, n = 5) for scissors traps and 0.22 and 0.38 Ns (mean = 0.30, SE = 0.03, n = 4) for Talpa traps. Clamping force ranged between 23.1 and 33.1 N (mean = 28.29, SE = 1.58, n = 5) for Duffus traps, 33.9 and 58.6 N (mean = 45.48, SE = 4.52, n = 5) for scissors traps and 67.5 and 86.7 N (mean = 79.25, SE = 4.36, n = 4) for Talpa traps. Overall there was wide variation in the forces produced by mole traps of different types and (among similar types) between manufacturers, with impact momentum varying approximately seven-fold and clamping force approximately four-fold across all of the traps measured.

We examined the effect of trap type and manufacturer on impact momentum and clamping force. First we tested two sub-sets of the data providing a balanced design between trap types (three manufacturers each producing scissors, Duffus and talpa (SDT group), and five manufacturers each producing both scissors and Duffus traps (SD group)) and then all manufacturers and trap types together (all traps). Both impact momentum and clamping force differed significantly among trap type for the SDT group (impact momentum F_2,87_ = 49.38 P<0.001, clamping force F_2,177_ = 894.57 P<0.001), the SD group (impact momentum F_1,98_ = 12.67 P<0.001, clamping force F_1,198_ = 272.82 P<0.001) and for all traps (impact momentum F_2,137_ = 57.47 P<0.001, clamping force F = _2,277_749.14 P<0.001). Overall, talpa traps produced the greatest and Duffus the weakest forces.

**Figure 4:**
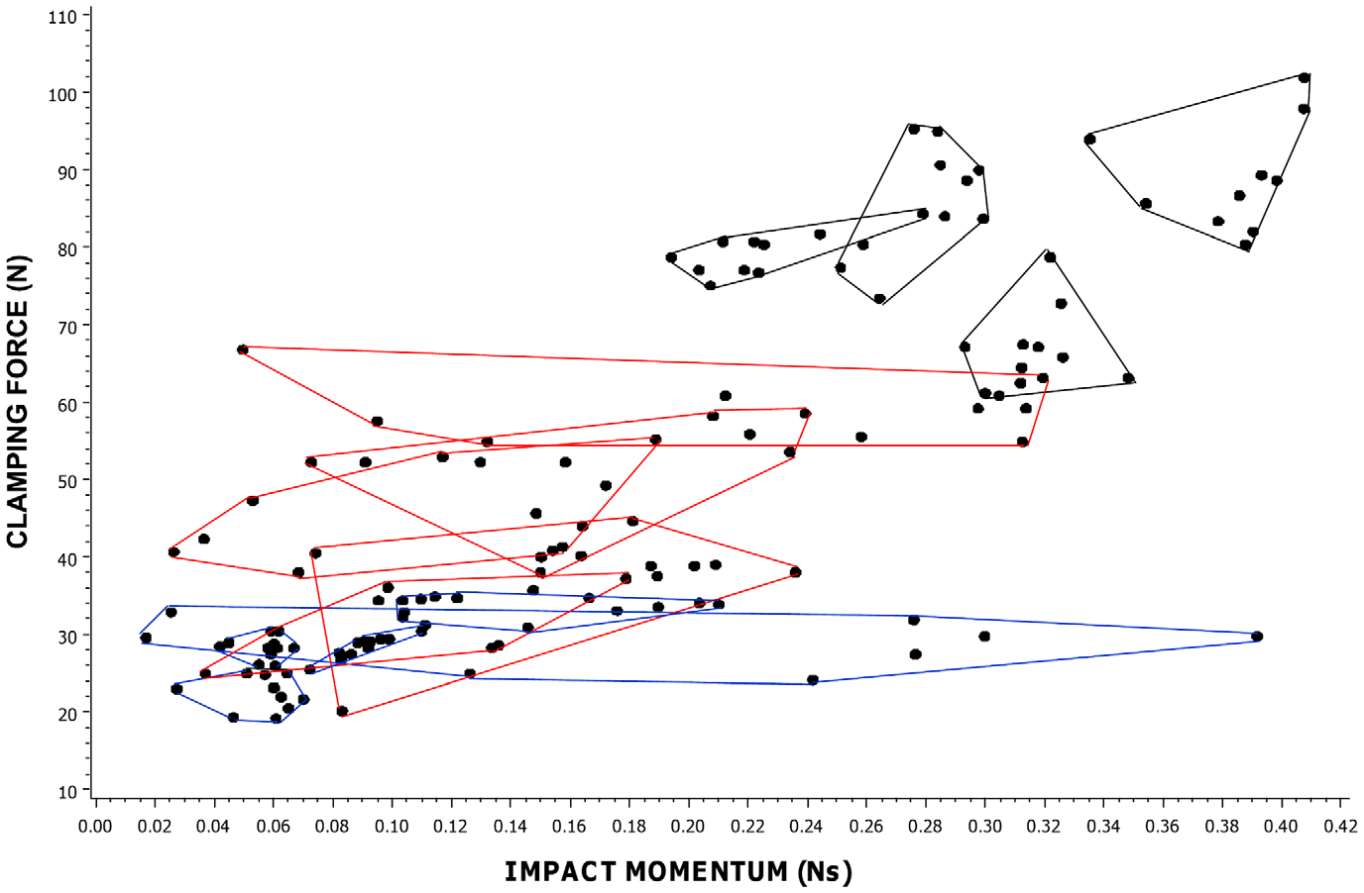
Raw data for impact momentum against clamping force in mole traps. Each point represents an individual trap and traps of the same trap type/manufacturer combination are enclosed within the same polygon. Trap types are: blue = Duffus, red = scissors, black = talpa.

There was a significant interaction between manufacturer and trap type for both impact momentum and clamping force for each of the groups (SDT group, impact momentum F_4,81_ = 14.28 P<0.001, clamping force, F_4,171_ = 54.05 P<0.001; SD group impact momentum, F_4,90_ = 10.20 P<0.001, clamping force, F_4,190_ = 46.41 P<0.001; all traps, impact momentum F = _6,126_10.05 P<0.001, clamping force F_6,266_ = 43.30 P<0.001), meaning that the pattern of forces among the trap types varied among manufacturer. Therefore we examined between-manufacturer variation in both measures for each trap type separately and found a significant difference between manufacturers in both impact momentum and clamping force for each trap type (scissors, impact momentum F_4,45_ = 3.43 P = 0.016, clamping force F_4,95_ = 64.42 P<0.001; Duffus, impact momentum F_4,45_ = 7.67 P<0.001, clamping force F_4,95_ = 55.83 P<0.001; talpa, impact momentum F_3,36_ = 103.84 P<0.001, clamping force F_3,76_ = 47.92 P<0.001). See Figure 4.

### Relationship between Trap Performance and Price in Mole Traps

Mole trap prices ranged between £1.95 and £10.85 across all types and manufacturers. Prices also varied widely within trap type and there was considerable price overlap between trap types (Table 6) with no significant difference in price between trap types (SDT group, F_2,6_ = 0.38 P = 0.697; SD group, F_1,8_ = 0.0 P = 0.967; all traps, F_2,11_ = 1.18 P = 0.344). There was however a price difference between manufacturers in both the balanced groups (SDT group, F_2,6_ = 14.53 P = 0.005; SD group, F_4,5_ = 14.04 P = 0.006).

**Table 6:** Mean, standard error, minimum and maximum prices paid for mole trap types.

	Price (£)				
	n	Mean	SE	Min	Max
**Scissors**	5	3.84	0.62	2.76	5.68
**Duffus**	5	3.88	0.69	1.95	5.68
**Talpa**	4	6.45	1.83	2.50	10.85

Prices are the mean of those paid for traps by four or five large manufacturers.

**Figure 5:**
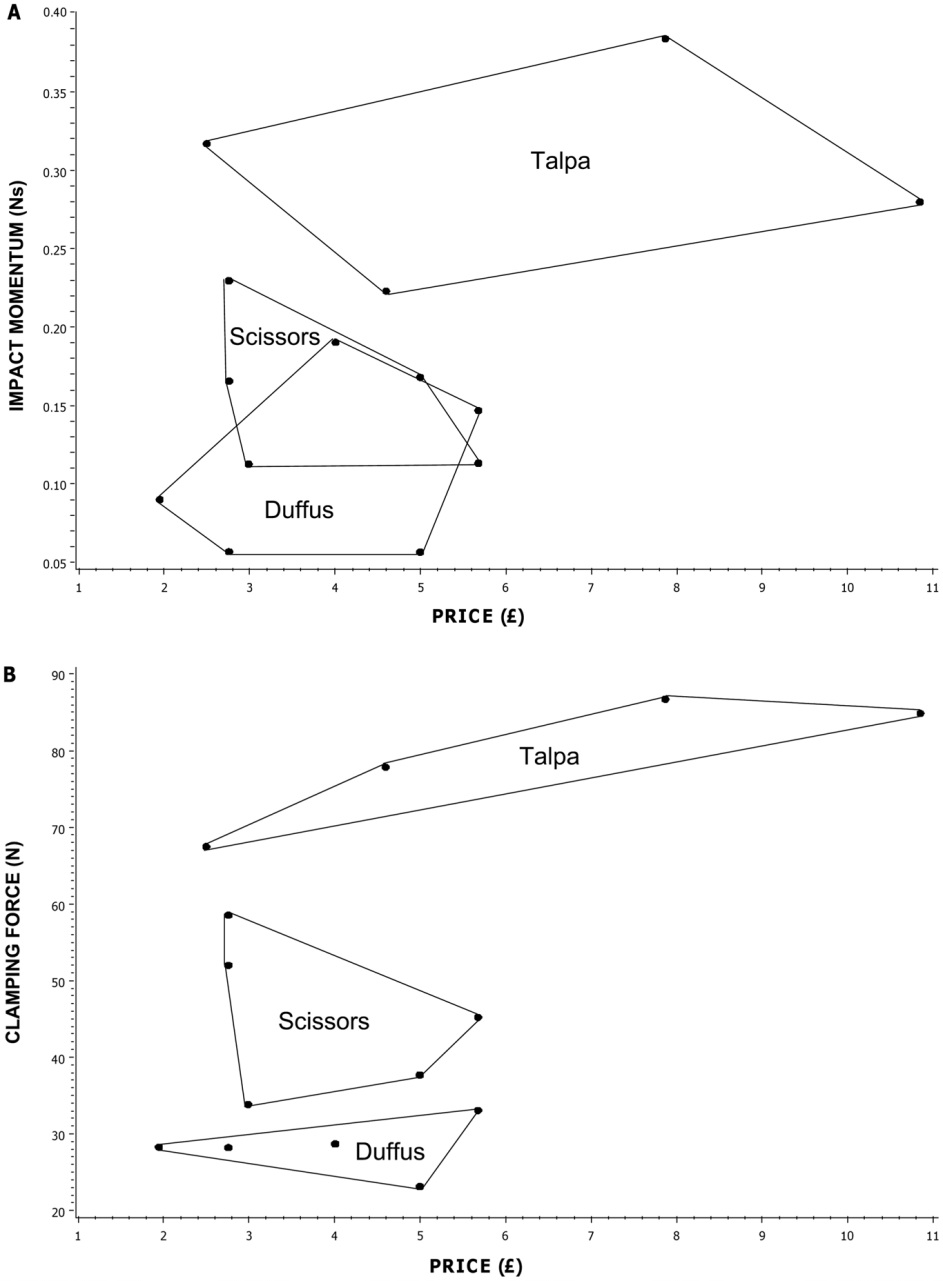
Price against mean mole trap performance. A) impact momentum; B) clamping force. Each point represents a trap type/manufacturer combination. Points for each trap type are enclosed in a polygon. There was a linear relationship between clamping force and price for talpa traps but no evidence for such a relationship for other trap types. There was no evidence of a linear relationship between impact momentum and price for any trap type.

There was no evidence of a linear relationship between price and trap performance for either scissors traps (impact momentum, F_1,3_ = 0.65, P = 0.480; clamping force, F = _1,3_0.42, P = 0.562) or Duffus traps (impact momentum, F_1,3_ = 0.26, P = 0.645; clamping force, F_1,3_ = 0.0, P = 0.959) (Figures 5A-B). While there was also no linear relationship for talpa traps between impact momentum and price (F_1,2_ = 0.03, P = 0.883), there was a positive linear relationship between clamping force and price (F_1,2_ = 19.15, P = 0.049); log (mean clamping force (N))  = 4.07807+ (0.16823× log (price (£)). The strongest mole trap in our mechanical tests overall was the second most expensive (a talpa) trap at £7.87. The strongest scissors trap produced a greater impact momentum than the same brand of talpa trap and cost just £2.76, joint cheapest among scissors traps. The two strongest Duffus traps cost £5.68 and £4.01, and the weakest cost £5.00, all more than the mean Duffus trap price (£3.88).

## Discussion

We measured impact momentum and clamping force in a range of break-back traps for rats and mice, and in several brands of three mole spring trap types. Without data relating mechanical trap performance to time to insensibility in trapped animals, or information regarding the possibility of different strike locations (except, in part, for moles (Baker et al, unpublished data, and [10]), we are unable to make judgements about the absolute levels of welfare impact associated with the traps tested. However by demonstrating several-fold variation in mechanical performance among traps for use with either rats, mice or moles, and considerable overlap in performance between mouse and rat traps, we have highlighted the potential welfare threat associated with their exemption from the UK approval process. We have also demonstrated clear evidence of scope for improving the standard of those traps available.

Impact momentum varied 6-8-fold, and clamping forces 4-5.5 fold, among traps for killing each species. Two types of rat trap and thirteen types of mouse trap fell in the range of overlap in impact momentum for the two species, while ten types of rat trap and nine types of mouse trap lay in the area of overlap in clamping force (Figure 1). Of course it might be that some traps were greatly over-engineered and that even the weakest traps tested for each species were sufficiently powerful to kill the target within an acceptable time. However given that the average weight of a brown rat *Rattus norvegicus* is more than 20 times that of a house mouse *Mus musculus* [9], the overlap in rat and mouse trap performance is cause for concern. Mechanical performance among mole traps varied among the three types (Duffus, scissors and talpa), with talpa traps producing the greatest forces overall, approximately twice those for scissors traps and three times those for Duffus traps. However, the mechanisms, and quite possibly associated strike locations, of the various mole trap types are different, and mechanical forces for different trap types may not translate into welfare impact in the same way. Differences between mole trap types may not, therefore, be cause for concern *per se*. However, forces produced by mole traps of the same type but different manufacturer varied by up to three times, and while all may be of an adequate standard this needs to be tested. The case of the long-accepted Fenn trap, which dramatically failed New Zealand’s new approval criteria, demonstrates the poor welfare situation that can occur when a trap is assumed to be of an adequate welfare standard. Eight of nine stoats trapped in approval tests remained conscious until euthanased after 5 minutes and the trials were stopped as a result [12]. Fenn traps are being replaced in New Zealand by new ‘DOC’ traps devised by the Department of Conservation there, and which follow National Animal Welfare Advisory Committee guideline (#09) (http://www.biosecurity.govt.nz/files/regs/animal-welfare/pubs/nawac/guideline09.pdf).

Our data highlight another potential issue with the current UK approval process, that of quality drift among new traps of a particular brand over time. Once approved, there is no monitoring and traps of a particular brand are assumed to be made to a consistent quality in perpetuity, whereas the manufacturer could potentially switch (knowingly or otherwise) to cheaper materials, components or manufacturing methods, with trap performance declining undetected. Our mole trap data showing widely varying mechanical performance, *between* different brands of the same trap type e.g. Duffus traps, demonstrate the potential for quality drift over time because outwardly the different brands look similar, but their performance is not. They may indeed have been the result of quality drift, if one manufacturer made a copy of another brand. To address this possible issue, manufacturers could be expected to produce mechanical test data from a sample of their approved traps, at specific intervals after approval is granted, for comparison with mechanical data submitted as part of their original approval request. Where a particular type of trap produces a consistent strike location in a given species, it might assist trap developers if they were given access to data on minimum mechanical standards that meet welfare thresholds. Another aspect of trap performance that is overlooked in all trap standards, agreements and guidelines is the deterioration of spring performance with use or time.

A further possible weakness of the UK approvals system relates to how tests are replicated. The 1999 ISO document on methods for testing killing traps makes the important point that sufficient replicates need to be tested to determine whether differences are statistically significant [4]. Currently the criteria for the spring traps approval process stipulate that 12 killing tests are required and that this is based on the Agreement on International Humane Trapping Standards. The agreement actually states that a minimum of 12 animals should be tested (http://www.canadainternational.gc.ca/eu-ue/assets/pdfs/eu25-en.pdf) but it is not clear whether these tests should be conducted using one trap 12 times, 12 traps once each, or in some other way. It has been suggested elsewhere that a complete evaluation of a particular trap might require lethal tests on 25 or more animals [13]. While the data for individual rat or mouse traps of some types, and for individual mole traps of certain type/manufacturer combinations, were tightly clustered, those for others exhibited a wide range of impact momentum measurements (e.g. individual rat traps of some types (Figure 2B), individual scissor mole traps by all manufacturers and individual Duffus mole traps of one brand (Figure 4)). Given the levels of variability identified between traps of the same type in this study, it is necessary to determine for each broad design of trap how many traps should be tested in order to gauge successfully the likely range of mechanical performance for a trap of that broad type. This applies to traps for all species.

It is not clear what the relative importance of impact momentum and clamping force might be for delivering a humane death, but each force in isolation can, in some circumstances, cause death. However, this depends on the species involved, the strike location and the forces applied [13]. Warburton and Hall’s study on possums [14] showed that the pathological effects of clamping forces in isolation were less severe than those of impact momentum alone. There is evidence from a number of animal species of a synergistic relationship between the forces in causing death ([14], and [15] cited therein). It seems likely that impact momentum will cause physical damage to the nervous system, blood vessels and organs, while clamping force will retain an injured animal in the trap, potentially causing asphyxiation or occlusion of blood vessels, and may increase damage if the animal struggles in the trap (P. West, Biomedical Services, University of Oxford, Pers. Comm.). Clamping force is also known to lessen any bounce-back of the striking components and according to Newcombe [16], cited in [5] “*provides an extra degree of insurance that a humane kill will be affected [sic]*”.

Zelin et al. [13] and Warburton and Hall [14] examined the separate and combined roles of impact momentum and clamping force in causing the death of anaesthetised mink, muskrats and raccoons, and of possums, respectively. These studies demonstrated that neither impact momentum nor clamping force thresholds were directly related to target species’ bodyweight and that these thresholds varied between strike locations within species. Minimum force thresholds to protect animal welfare can not therefore be extrapolated easily between species or strike locations. For these reasons, we do not aim to comment on the possible relative humaneness of the traps tested, should they hit an animal at strike locations other than the chest – our chosen strike location. (It would be possible to model the effect of different strike locations [i.e. different body thicknesses] on clamping force and impact momentum and it may be that the relative humaneness of our trap types, and brands, might change subtly with strike location). We restrict our comments and conclusions to demonstrating the relative effects of trap types and brands, and yet anticipate that a similar variation in trap performance might be observed if we were to test alternative strike locations. Nevertheless, the actual welfare impact of the traps tested in this study can only be determined by killing tests on the species concerned.

Trap-opening angle and spring type were important predictors of the mechanical forces produced by rat and mouse traps in this study. In general, impact momentum was positively, and clamping force negatively, related to opening angle (although the pattern was slightly different for impact momentum in mouse traps, with the largest impact momentum occurring in the mid-angle category, most likely because only one trap of nine in the wide angle category had a DPEG spring and this produced a low measurement). These patterns also occurred within each of the four spring types. Similar relationships were observed by Warburton [17] who wrote, of two of the traps tested in this study, “*The Snap-E rat trap has greater clamping force than the Victor trap and therefore may be effective against larger stoats. However, its impact momentum is likely to be less than the Victor because the striking bar only travels through 90* [measured at 80 in this study] *degrees before impact, in contrast to the Victor’s striking bar that travels through 180 degrees before impact*”. Spring type was also a useful predictor of mechanical performance. Traps with DPEG springs produced the greatest forces in rat and mouse traps, while the smallest forces were produced either by JAW springs (both forces in rat traps and impact momentum in mouse traps) or PULL springs (clamping force in mouse traps). Our findings regarding opening angle and spring type are illustrated by some examples among traps in the replicated set. Those rat traps with the largest angles and DPEG springs (see Rd and Rf in Table 1 and Figure 1) scored highly on impact momentum, while that with the smallest angle and a JAW spring scored lowest (Ra). The rat and mouse traps with the smallest angle and a DPEG spring (Rb and Ma) scored highest for clamping force.

Traditional break-back rodent traps consist of a flat wooden base, with a PEG or DPEG spring and an opening angle approximating 180 degrees (e.g. Figure S2A), this maximising the impact momentum delivered to the target animal on contact. While this type of trap apparently remains popular and relatively cheap, a wide range of largely plastic rodent traps have become available. These are often promoted on the grounds that they are easy to set and hygienic to use (the carcase can be released into a bin without touching it). However a default feature of this type of trap is a smaller opening angle, the smallest in this study being 45 degrees, creating a distance between the jaws, when set, of little over 3cm. Traps with acute opening angles in our study incorporated a variety of spring types including DPEGs and the generally weaker JAW and PULL springs. An advantage of the smaller angle seems to be that a greater clamping force is produced, because the spring is less unwound at the point of impact. However, as well as a stronger clamping force, a smaller opening angle produces a smaller impact momentum, because the distance travelled - and therefore terminal velocity of the striking components - is reduced. There is therefore a potential trade-off between impact momentum and clamping force and depending on the relative importance of the two in causing a quick kill, it could be a mid-angle trap that delivers the best welfare outcome, but this needs to be determined through killing tests.

In terms of pure mechanics, impact momentum is generated by the product of the mass of striking elements of the trap and the velocity they reach when they make contact with an animal. Because velocity increases with spring stiffness, it is theoretically possible to compensate for a narrow trap opening angle by using a stronger spring to generate impact momentum values comparable to existing wide angle traps. This would also give the small angle traps a proportionally greater clamping force, but might prove impractical in terms of trap-setting or design. For example, increasing the spring power may not be possible for some traps, either because the trap frame distorts, or the trigger sensitivity changes and the trap may not set or trigger correctly. So it may not be the general design of this new generation of narrow opening traps that is inherently weak, just the strength and/or type of the spring – something that may or may not be remedied by the manufacturers. However, other elements of design, for example, quality of the spring mechanism, make this relationship less clear – again something that could only be tested through experimentation. The effect of a strong spring, or wide opening angle could be dampened by heavy striking elements or resistance in hinges. The only way to assess these types of influences would be to deconstruct traps and carry out further, and far more laborious, tests. It is simpler to consider the forces measured here and whether any unexpectedly weak traps could deliver greater forces by using a stronger spring. Our results must be considered with it in mind that certain spring types were used in certain types of trap, with particular opening angles and that our conclusions are based on the trap types studied here.

Small opening angle trap designs might offer a welfare benefit in that they should be more species specific, i.e. less likely to trap a larger species or larger body part, e.g. the paw of a larger animal. They should be less likely to be fouled in operation, e.g. by the travel of the striking components being hindered by obstacles, so increasing the chance of a clean strike. Using better quality DPEG springs in such a small opening angle trap, might increase the impact momentum produced, while increasing an already large clamping force and retaining the advantages of a modern plastic trap. We hope our findings might help both in the development of optimum spring traps as well as assisting consumers in identifying more powerful rat and mouse traps.

Price was not a reliable indication of mechanical performance in rat or mouse traps, nor in mole traps, although more expensive talpa traps produced greater clamping forces, but this was based on a small sample (n = 5), and so may not be reliable. Clearly one does not necessarily get what one pays for in terms of trap performance. As part of their recent report to the EU, FERA conducted a survey on public attitudes to trapping within the EU, and 71% of current trappers said they were not prepared to pay more for a trap that had been tested and approved (Talling and Inglis 2009). However, this may not be representative of trappers in the UK as the majority of survey respondents were from the European continent and fewer than 3% from the UK. Nevertheless, trap development and testing are likely to incur costs for manufacturers, but given that in general we found no relationship between price and trap performance, producing a more powerful trap *per se* ought not automatically be more expensive.

FERA’s public attitudes survey revealed that while the public accepted that human and/or environmental needs could justify the killing of animals, they also believed that the welfare of trapped animals was important. As a result they wanted trapping within the EU to be regulated by legislation covering all species that could legally be trapped and the traps used to be tested and approved by an independent institute using clearly defined animal welfare guidelines (Talling and Inglis 2009). Where traps are subjected to killing tests under the current spring traps approval process in England, the time to irreversible unconsciousness from initial strike is determined by loss of palpebral and corneal reflexes. If 80% of 12 tests cause irreversible unconsciousness within 300 seconds (5 minutes), then the trap is recommended for addition to the Spring Traps Approval Order for each species for which this is achieved (Defra, Pers. Comm.). These criteria are based on the Agreement on International Humane Trapping Standards (AIHTS) (http://www.canadainternational.gc.ca/eu-ue/assets/pdfs/eu25-en.pdf). Additional species may be included in the approval if expert opinion is that data from the test species indicate the trap would be as humane for these additional species.

When respondents in Talling and Inglis’ (2009) survey were asked about the maximum acceptable period between trapping and the “*unconsciousness and death*” of the captured animal, 29% said death should be instantaneous (zero seconds), 26% opted for a maximum of 30 seconds and only 6% felt that the 300 seconds period, contained in the AIHTS, was acceptable. Subsequently Talling and Inglis (2009) proposed Improved Standards to increase the welfare of trapped animals. These involve three Welfare Categories of trap, differing in times to irreversible unconsciousness (TIU) of trapped animals (Table 7). Talling and Inglis (2009) suggested that where traps in different categories were available, only those in the highest welfare category should be used, in order to encourage the improvement of trap standards. Our results suggest that rat, mouse and mole welfare in the UK might benefit from adopting such a system.

**Table 7:** Welfare Categories proposed in FERA’s recent review of trapping standards (Talling and Inglis 2009).

Welfare Category	Requirements regarding time to irreversible unconsciousness (TIU)
**A**	≥80% of trapped animals have a TIU ≤30 seconds, ≥90% have a TIU ≤180 seconds
**B**	≥80% of trapped animals have a TIU ≤180 seconds, ≥90% have a TIU ≤300 seconds
**C**	≥80% of ≥12 animals tested have a TIU ≤300 seconds (current AIHTS standard)

FERA = The Food and Environment Research Agency. AIHTS = Agreement on International Humane Trapping Standards.

Since the current approval criteria (for traps that need approval) require killing-tests, we believe there is scope for designing animal analogues (or ‘Trap-test dummies’), i.e. standard animal models, to be used in place of live animals in trap tests. Initial tests would be required to establish if a given threshold of damage to the analogue was equivalent to a lethal strike from a trap at different strike locations (according to a standard for time to reach irreversible unconsciousness), but beyond this, the analogue could replace live tests on anaesthetised animals (where these are considered valuable), thereby removing the ethical objections, and reducing the considerable cost, associated with repeated killing-tests. Not only would an analogue provide data on whether acceptable mechanical thresholds were being met, but it would also provide data on the specific performance of each new variant of trap tested – effectively adding to the knowledge base. We suggest this idea could be taken forward as a desk study initially to establish the feasibility of the idea, but also as a means of taking on development partners.

### 

#### Summary

In 1951, the Committee on Cruelty to Wild Animals felt that neither mole spring traps nor rat or mouse break-back traps caused unnecessary suffering [1], although it seems there was no evidence for this, but rather no evidence against. Indeed, in support of their assertions about rat traps, the Committee included statements about the rat’s pest status and that its control and destruction were considered essential, neither of which ought to have any bearing on the need for welfare standards in managing the species. It is likely that the exemption of break-back traps for rats and mice, and mole spring traps from the UK approval process, has hindered improvements in welfare standards. Today it is hard to think of a valid reason for excluding from approval any traps for these species, particularly given the proliferation of trap types and brands available, including the influx of plastic rat and mouse traps to the market, with their small opening angles and in some cases weak types of spring. In addition to the traps tested here there are doubtless others available, particularly on the internet and from overseas, e.g. China, including many unbranded break-back traps. To further complicate the issue, one UK company told us that they packaged the same unmarked mouse break-back traps for different companies.

In summary, the welfare of rats, mice and moles should be taken into account, as it is for other species, when designing traps for killing them. If traps for these species are to be included in the approval process, each type should have to meet the same standards as new traps; none should be approved automatically on the basis of their long-standing or prior existence. This will involve killing trials in the first instance to determine threshold impact momentum and clamping force values for these species and in the case of moles for the different trap types. We agree with Talling and Inglis (2009) that spring traps should require approval for all trapped species and that a tiered welfare system could, particularly in the case of break-back traps for rats and mice, and mole spring traps, stimulate an ongoing improvement in trap welfare standards for these species.

## Supporting Information

Figure S1 **Rat and mouse trap types tested.** These comprised 18 rat traps (1-18, top two rows) and 23 mouse traps (1-23, bottom two rows). Numbers relate to labels shown in Table S1.
(JPG)

Figure S2 **Spring types identified in rat and mouse traps.** A) peg (PEG); B) double peg (DPEG); C) jaw (JAW); D) pull (PULL).
(JPG)

Figure S3 **Measurement of trap opening-angle shown with a mouse trap in the set position.**
(JPG)

Figure S4 **Mole trap types tested.** A) Scissors; B) Duffus; C) Talpa.
(JPG)

Figure S5 **Dynamic load cell in aluminium jig (with scissors trap).**
(JPG)

Figure S6 **Raw data for impact momentum against clamping force in mouse and rat traps.** A) mouse traps; B) rat traps. Each point represents a separate measurement and measurements from the same trap are enclosed within a polygon. Points marked Ma-f (labelled blue) and Ra-f (labelled red) are trap types in the mouse and rat replicated sets respectively, and are identified on Figures 1 and 2.
(PDF)

Table S1 **Rat and mouse break-back trap types tested in the study.** A) rat traps; B) mouse traps. Numbers relate to labels shown in Figure S1. Traps are presented in alphabetical order.
(PDF)

Table S2 **The number of rat and mouse trap types and individual traps of each type studied.**
(PDF)

Table S3 **Sample sizes of rat and mouse trap types in each angle category/spring type combination.**
(PDF)
