# Supplementary figures and images for: Mechanical Performance of Rat, Mouse and Mole Spring Traps, and Possible Implications for Welfare Performance
Source: PLoS One. 2012 Jun 29;7(6):e39334. doi: 10.1371/journal.pone.0039334 (PMC3387155; doi:10.1371/journal.pone.0039334)

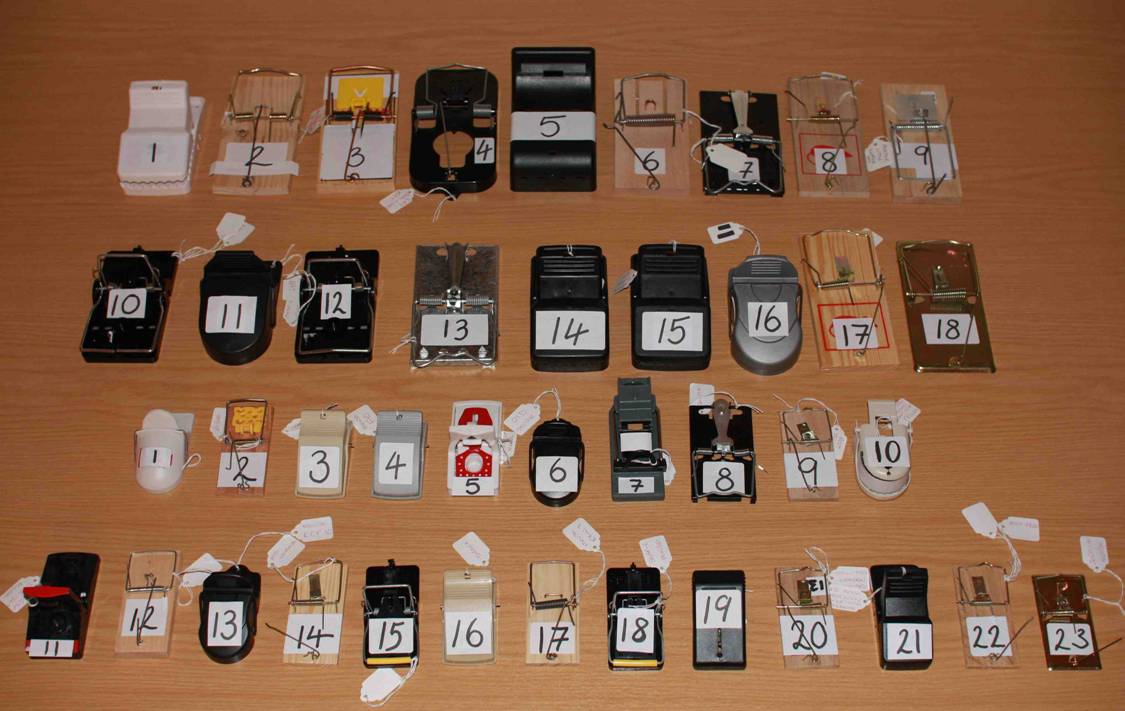

Supplement: Figure S1 — Rat and mouse trap types tested. These comprised 18 rat traps (1-18, top two rows) and 23 mouse traps (1-23, bottom two rows). Numbers relate to labels shown in Table S1. (JPG) [file pone.0039334.s001.jpg]

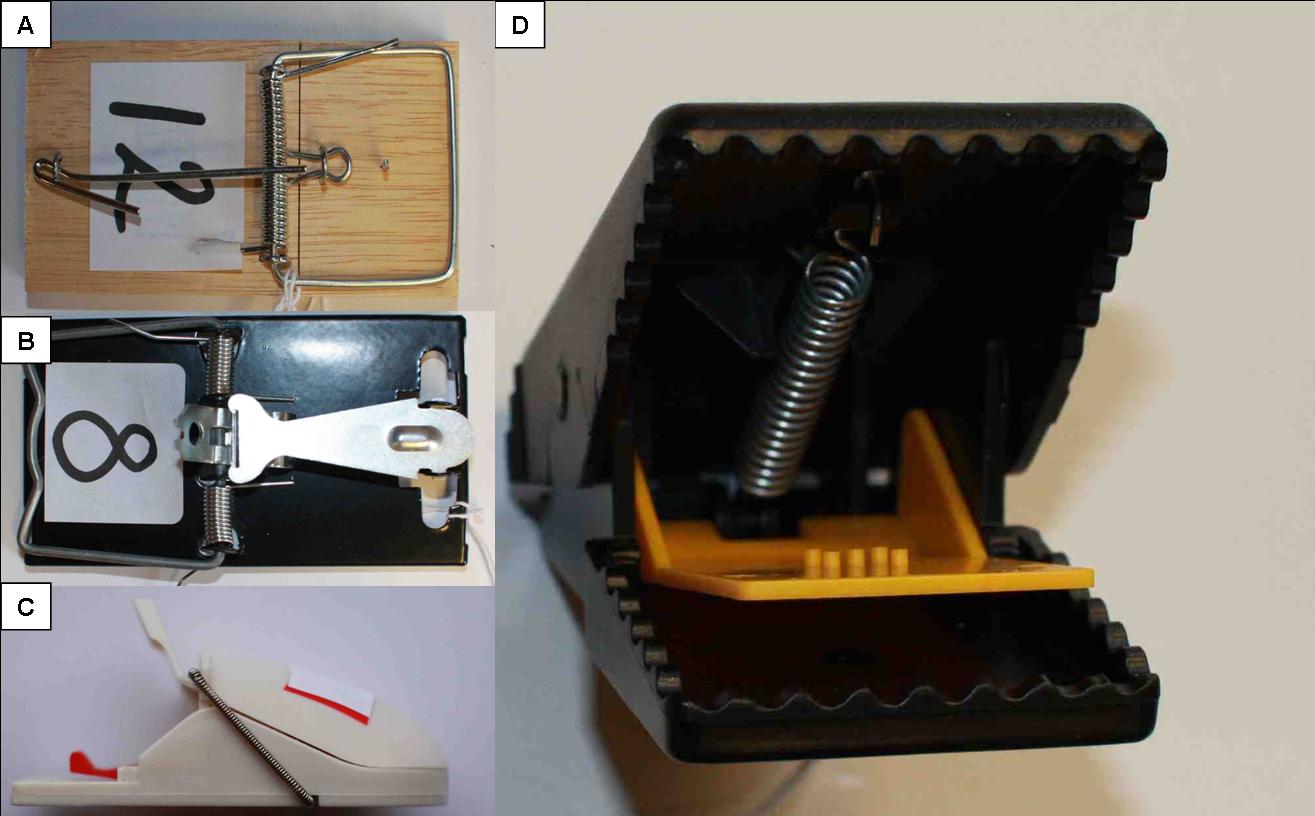

Supplement: Figure S2 — Spring types identified in rat and mouse traps. A) peg (PEG); B) double peg (DPEG); C) jaw (JAW); D) pull (PULL). (JPG) [file pone.0039334.s002.jpg]

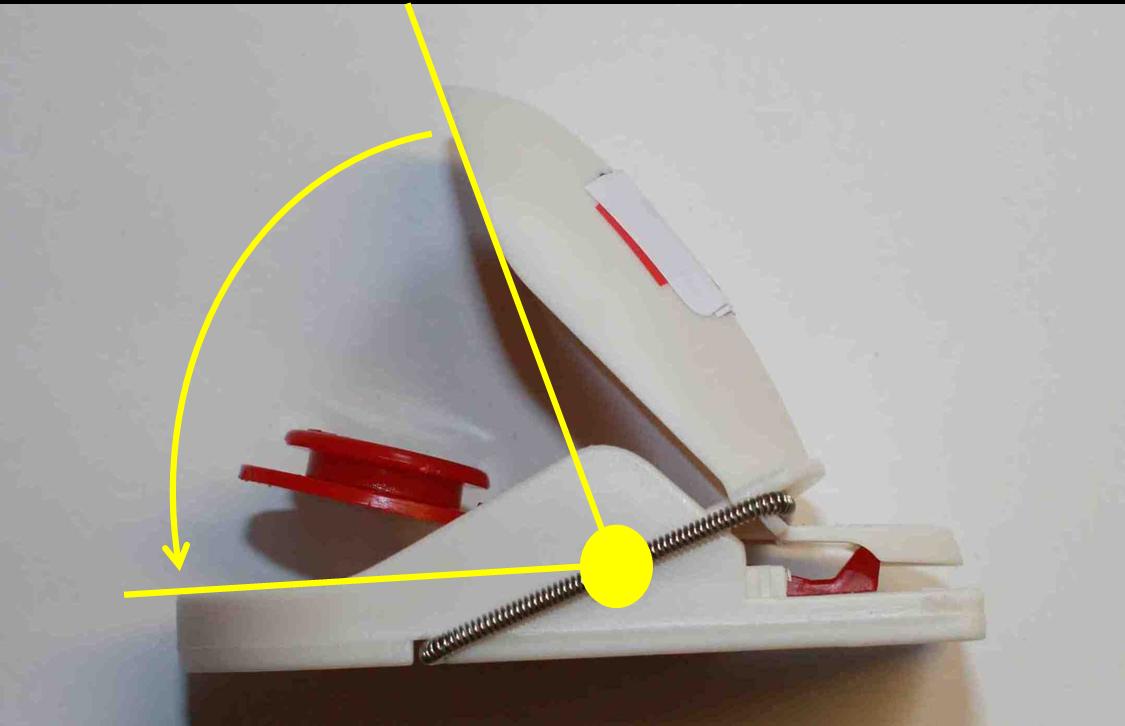

Supplement: Figure S3 — Measurement of trap opening-angle shown with a mouse trap in the set position. (JPG) [file pone.0039334.s003.jpg]

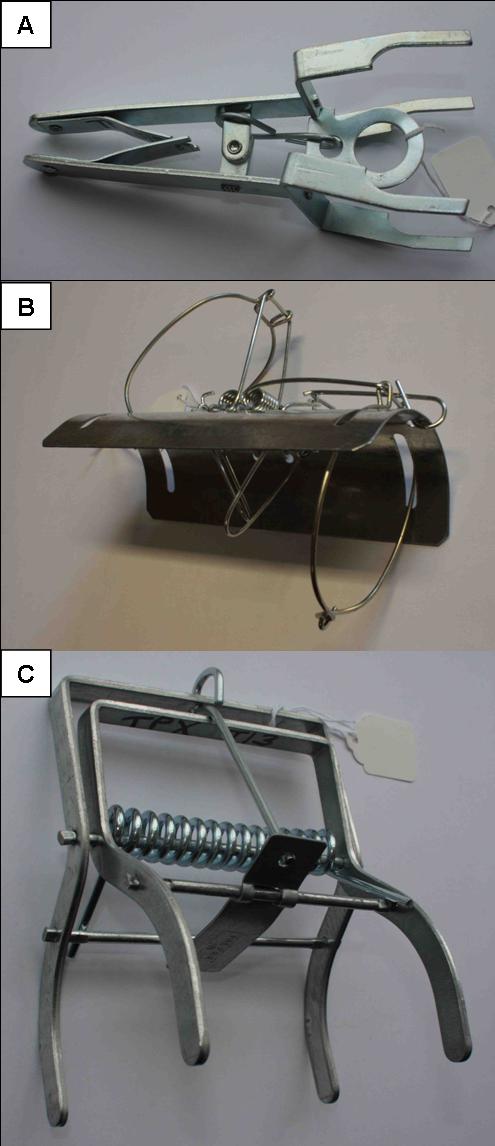

Supplement: Figure S4 — Mole trap types tested. A) Scissors; B) Duffus; C) Talpa. (JPG) [file pone.0039334.s004.jpg]

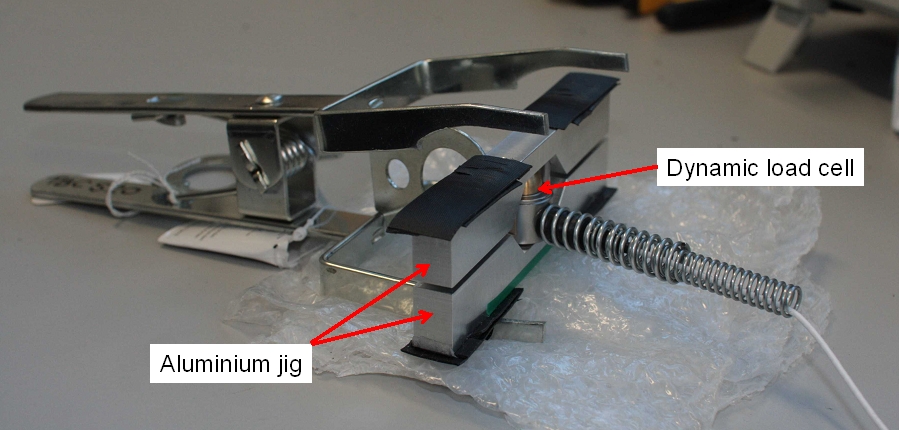

Supplement: Figure S5 — Dynamic load cell in aluminium jig (with scissors trap). (JPG) [file pone.0039334.s005.jpg]

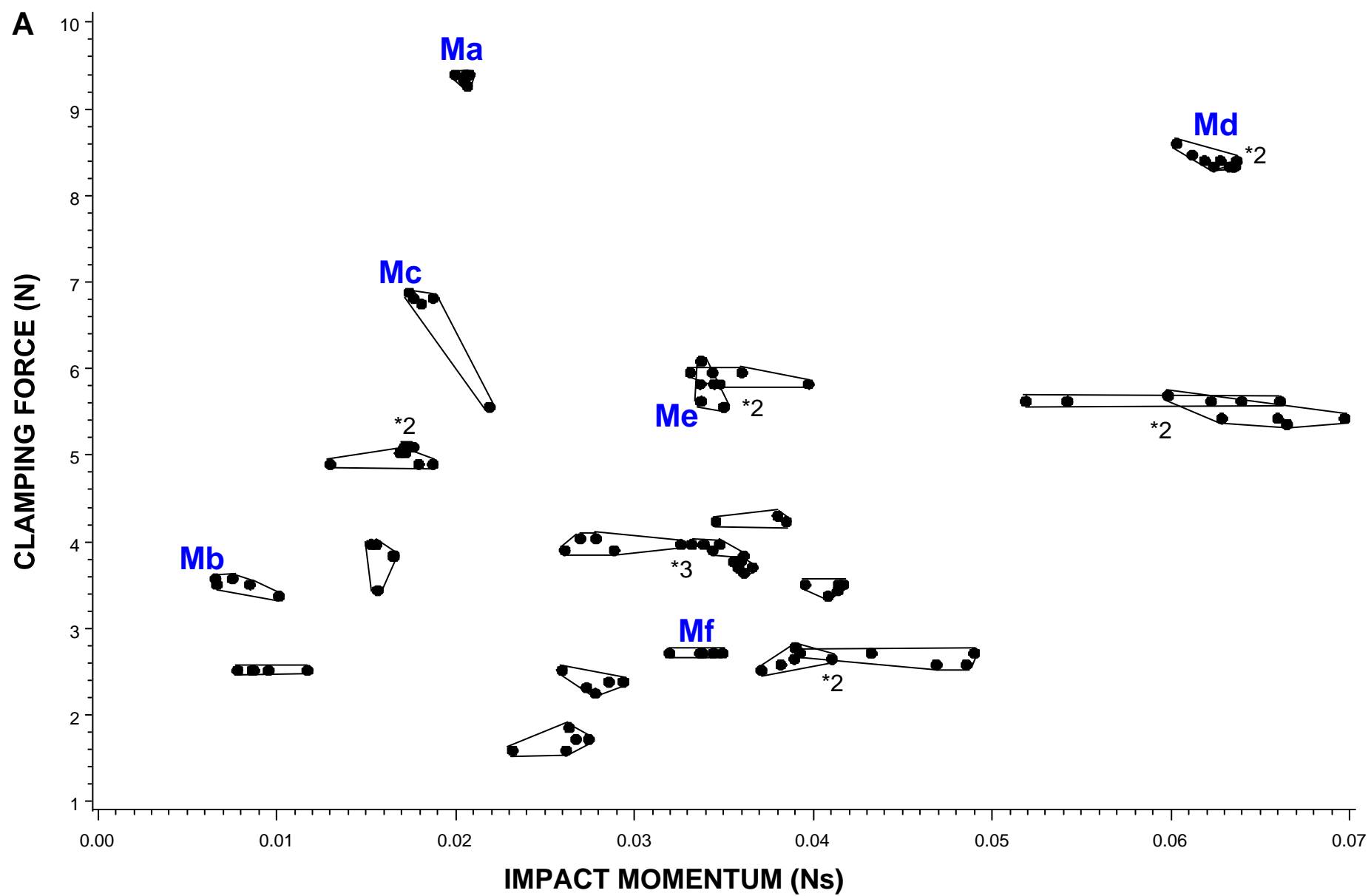

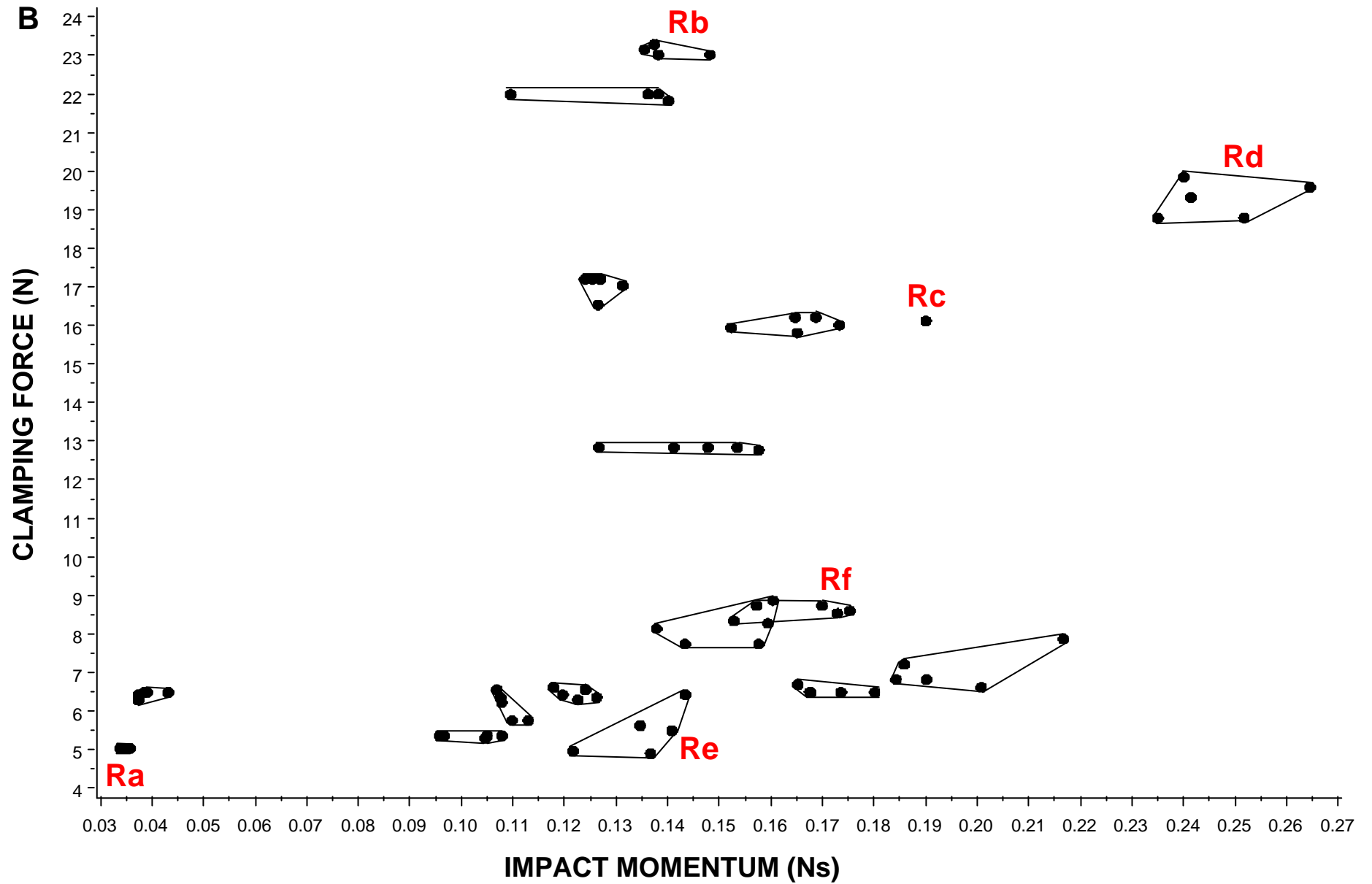

Supplement: Figure S6 — Raw data for impact momentum against clamping force in mouse and rat traps. A) mouse traps; B) rat traps. Each point represents a separate measurement and measurements from the same trap are enclosed within a polygon. Points marked Ma-f (labelled blue) and Ra-f (labelled red) are trap types in the mouse and rat replicated sets respectively, and are identified on Figures 1 and 2. (PDF) [file pone.0039334.s006.pdf]
