# Supplementary material for: Mechanical Performance of Rat, Mouse and Mole Spring Traps, and Possible Implications for Welfare Performance
Source: PLoS One. 2012 Jun 29;7(6):e39334. doi: 10.1371/journal.pone.0039334 (PMC3387155; doi:10.1371/journal.pone.0039334)
Supplement: Table S1 — Rat and mouse break-back trap types tested in the study. A) rat traps; B) mouse traps. Numbers relate to labels shown in Figure S1. Traps are presented in alphabetical order. (PDF) [file pone.0039334.s007.pdf]

**A) Rat trap types**	**1**	Big Cheese Quick Click Rat Trap
	**2**	Big Cheese Pro-Strength Pre-Baited Rat Trap
	**3**	Bayer Garden (Victor) Professional Rat Trap
	**4**	CAT Safe & Sure Rat Trap
	**5**	Fito Heavy Duty Prebaited Easy Set Plastic Rat Trap
	**6**	Fito Wooden Rat Trap
	**7**	Procter Pest-Stop Easy Setting Metal Rat Trap
	**8**	Pest Police Rat-Trap
	**9**	Procter Little Nipper
	**10**	Rentokil Advanced Rat Trap
	**11**	Rolson Rat Trap
	**12**	Snap-E Rat Trap
	**13**	Self-set Metal Rat Trap
	**14**	Tom Cat Rat Snap Trap
	**15**	Trapper T-Rex Rat Snap Trap
	**16**	Yeoman Speed-Set Rat Trap
	**17**	Yeoman Traditional Rat Trap
	**18**	Zero-In Metal Rat Trap
**B) Mouse trap types**	**1**	Big Cheese Quick Click Prebaited Mouse Trap
	**2**	Big Cheese Pro-Strength (Easy To Set) Mouse Trap
	**3**	Bayer Garden (Victor) Advanced Mouse Trap
	**4**	Fito Pre-Baited Easy-Set Mouse Trap
	**5**	Home Defence Press N Set Mouse Trap
	**6**	Jak Mouse Trap
	**7**	Procter Pest-Stop Advanced (Quick Kill) Mouse Trap
	**8**	Procter Pest-Stop Easy Setting Metal Mouse Trap
	**9**	Pest Police Mouse-Trap
	**10**	Procter Pest-Stop Pre-Baited (Plastic) Snap Trap
	**11**	Procter Pest-Stop Sure-Set Mouse Trap
	**12**	Procter Little Nipper
	**13**	Raco Mouse Killer Snap Trap
	**14**	Raco Wooden Mighty Mouse Trap
	**15**	Rentokil Advanced Mouse Trap
	**16**	Rentokil Quick-Set Mouse Trap
	**17**	Rentokil (Wooden) Mouse Trap
	**18**	Snap-E Mouse Trap
	**19**	Tom Cat Mouse Snap Trap
	**20**	Tool-Tech Wooden Mouse Trap
	**21**	Trapper T-Rex (Mini Rex) Extra Strength Mouse Snap Trap
	**22**	'UK Brand' Mouse Snappa
	**23**	Zero-In Metal Mouse Trap
