# Supplementary material for: Mechanical Performance of Rat, Mouse and Mole Spring Traps, and Possible Implications for Welfare Performance
Source: PLoS One. 2012 Jun 29;7(6):e39334. doi: 10.1371/journal.pone.0039334 (PMC3387155; doi:10.1371/journal.pone.0039334)
Supplement: Table S2 — The number of rat and mouse trap types and individual traps of each type studied. (PDF) [file pone.0039334.s008.pdf]

| A) **Rat traps** | **No. of trap types** | **No. of replicates** | **Total no. of traps** |
|---|---|---|---|
| Non replicated-set | 12 | 1 | 12 |
| Replicated set | 6 | 15 | 90 |
| Total | 18 | | 102 |
| B) **Mouse traps** | **No. of trap types** | **No. of replicates** | **Total no. of traps** |
| Non replicated-set | 17 | 1 | 17 |
| Replicated set | 6 | 15 | 90 |
| Total | 23 | | 107 |
