# Supplementary material for: Mechanical Performance of Rat, Mouse and Mole Spring Traps, and Possible Implications for Welfare Performance
Source: PLoS One. 2012 Jun 29;7(6):e39334. doi: 10.1371/journal.pone.0039334 (PMC3387155; doi:10.1371/journal.pone.0039334)
Supplement: Table S3 — Sample sizes of rat and mouse trap types in each angle category/spring type combination. (PDF) [file pone.0039334.s009.pdf]

	A) Rat traps			B) Mouse traps		
	**Angle cat 1**	**Angle cat 2**	**Angle cat 3**	**Angle cat 1**	**Angle cat 2**	**Angle cat 3**
**PEG**			5			8
**DPEG**	5		5	6	2	1
**JAW**	2			3		
**PULL**	1			1	2	
